# Wernicke encephalopathy in non-alcoholic patients following gastrointestinal procedures: a systematic review

**DOI:** 10.3389/fsurg.2026.1802386

**Published:** 2026-06-30

**Authors:** Hala Abu-Qasem, Husna Irfan Thalib, Zainab Shoeb Ghazi, Reem Tageldin, Yara Alras, Hanin Faisal, Laiba Khan, Abderrahman Ouban, Mounzer Yassin-Kassab

**Affiliations:** 1 College of Medicine, Alfaisal University, Riyadh, Saudi Arabia; 2 General Medicine Practice Program, Batterjee Medical College, Jeddah, Saudi Arabia; 3 Department of Neurology, University of Tennessee and Erlanger Hospital, Chattanooga, TN, United States

**Keywords:** gastrointestinal surgery, non-alcoholic, postoperative complications, thiamine deficiency, vomiting, Wernicke encephalopathy

## Abstract

**Background:**

Wernicke encephalopathy (WE) is a serious neurological disorder often linked to alcohol use but can also occur under non-alcoholic conditions, particularly following gastrointestinal (GI) procedures. Existing reviews predominantly focus on bariatric surgery, leaving significant gaps regarding other major GI procedures.

**Objective:**

This study aimed to systematically review and synthesize evidence on the incidence, clinical characteristics, diagnostic methods, management strategies, and outcomes of WE in non-alcoholic adults following gastrointestinal procedures.

**Methods:**

A PRISMA-compliant systematic review was conducted. PubMed, Scopus, Web of Science, and Embase were searched for studies published between 2000 and 2025 that reported Wernicke encephalopathy in non-alcoholic adults following gastrointestinal procedures. Two reviewers independently screened and selected studies. Quality assessment was performed using the Newcastle–Ottawa Scale and Joanna Briggs Institute checklist.

**Results:**

The review included 13 studies involving 1,036 patients. Diverse procedures, including bariatric, oncologic, and emergency gastrointestinal surgeries, were associated with WE, with vomiting identified as the most common precipitating factor. The classical triad of confusion, ataxia, and oculomotor dysfunction appeared in only a minority of patients, while most patients presented with atypical features such as cognitive deficits and polyneuropathy. Magnetic resonance imaging demonstrated characteristic changes in the thalami, mammillary bodies, and periaqueductal gray in confirmed cases. Rates of mortality varied considerably across studies, ranging from 0.3% in bariatric cohorts to 40% in oncologic patients. Early intravenous thiamine was associated with favorable outcomes, although dosing protocols varied considerably across studies.

**Conclusion:**

WE following gastrointestinal procedures presents significant diagnostic challenges. Prophylactic thiamine supplementation should be strongly considered in high-risk patients, particularly those with malnutrition, prolonged fasting, or persistent vomiting.

**Systematic Review Registration:**

https://www.crd.york.ac.uk/PROSPERO/view/CRD420251164201, PROSPERO CRD420251164201.

## 1 Introduction

Wernicke encephalopathy (WE) is a serious and potentially life-threatening acute neurological disorder caused by thiamine (vitamin B1) deficiency. Although alcohol abuse is the most common cause, it can also occur in non-alcoholic settings such as prolonged starvation and gastrointestinal (GI) surgery (1). WE affects both the central and peripheral nervous systems and typically presents with a triad of impaired eye movements (with nystagmus), ataxia, and altered mental status (2). However, this triad occurs in only about 10% of cases, making diagnosis challenging (3).

Non-alcoholic patients often present with different clinical signs and imaging findings compared with alcoholic cases, further complicating diagnosis (4). Autopsy studies report WE in 0.4%–2.8% of cases, yet it is frequently missed during life. It remains undiagnosed in approximately 68% of individuals with alcoholism and up to 94% of non-alcoholic patients (5). If untreated, WE progresses to Korsakoff syndrome (KS) in nearly 80% of cases, characterized by irreversible memory deficits and psychosis, and it may develop even without preceding acute WE symptoms (1). Because of overlapping features, WE and KS are collectively termed Wernicke–Korsakoff syndrome (WKS). Early recognition and prompt intravenous thiamine treatment are therefore critical to reduce morbidity and mortality (6).

Thiamine is a water-soluble vitamin essential for carbohydrate metabolism and normal brain function (7). It also helps maintain membrane integrity and osmotic gradients. A significant level of thiamine is stored in body tissues as thiamine diphosphate (TDP) (8). Adults require 1–2 mg/day, depending on carbohydrate intake; an increased carbohydrate load raises thiamine demand and may precipitate deficiency. Deficiency can result from inadequate intake, impaired absorption, excessive loss, or reduced conversion to its active form (9).

As thiamine levels decrease, glucose metabolism becomes impaired, leading to mitochondrial dysfunction, lactic acidosis, oxidative stress, and disrupted neurotransmitter and DNA synthesis (8). Acute WE is treated with intravenous thiamine hydrochloride, along with correction of underlying causes and nutritional deficiencies. However, delayed or inadequate treatment may result in progression to KS (10).

The diagnosis of WE is primarily clinical but may be supported by investigations such as brain magnetic resonance imaging (MRI), routine blood tests, and thiamine levels. Plasma thiamine levels are often unreliable, as they may appear normal because of timing and uneven tissue distribution. More accurate tests, including erythrocyte transketolase activity and the thiamine pyrophosphate effect, are rarely used because of cost and limited availability (11). The Caine criteria aid clinical diagnosis and require at least two of the following: dietary deficiency, oculomotor abnormalities, cerebellar dysfunction, and altered mental status or memory impairment. While MRI [especially fluid-attenuated inversion recovery (FLAIR) sequences] and whole blood thiamine levels can support diagnosis, serum levels are less reliable. Given these limitations, empirical intravenous thiamine should be initiated whenever WE is suspected, even before confirmation (12).

MRI is particularly useful in atypical or non-alcoholic cases. Typical findings include symmetrical T2-weighted and FLAIR hyperintensities in the periaqueductal area, medial thalami, mammillary bodies, tectal plate, and periventricular regions of the third and fourth ventricles (14). Atypical findings may involve the cerebellum, vermis, cranial nerve nuclei, red nuclei, dentate nuclei, caudate nuclei, corpus callosum, and cerebral cortex (15). Computed tomography (CT) is generally insensitive and often normal, limiting its diagnostic value compared with MRI (13).

Gastrointestinal surgery increases the risk of thiamine deficiency through malabsorption, dietary restriction, and increased metabolic demand. Non-alcoholic WE accounts for 20%–50% of cases and is frequently associated with GI surgery, chronic malnutrition, and parenteral nutrition (16). All bariatric procedures can lead to WE (17), and similar risks apply to other major GI surgeries. Procedures involving the duodenum and proximal jejunum carry higher risk, as these are key sites of thiamine absorption.

Diagnosing WE in postsurgical patients is especially challenging when classical symptoms are absent (18). WE typically develops 4–12 weeks postoperatively, with 79.1% of cases occurring within 6 months (17). Vomiting, the most common precipitating factor (87.3% of cases), reduces intake and accelerates thiamine depletion (17, 18). Prophylactic thiamine supplementation should therefore be considered in patients undergoing major GI surgery, particularly for those with preoperative malnutrition or postoperative feeding difficulties.

Despite increasing awareness, the existing literature on WE after GI surgery remains limited. A majority of published evidence and prior reviews focus on bariatric surgery (19, 20), leaving gaps regarding other GI surgeries. This review addresses that gap by examining WE following diverse gastrointestinal surgeries—including oncologic and emergency procedures—in non-alcoholic adults, synthesizing evidence from 2000 to 2025. Through this, we aim to improve early detection, prevention, and management of this potentially reversible condition.

## 2 Methods

This systematic review followed the Preferred Reporting Items for Systematic Reviews and Meta-Analyses (PRISMA) statement (21) to ensure comprehensive reporting and transparency. Although PRISMA guidelines were followed, the included studies were predominantly observational and descriptive in nature, which limits the applicability of certain quantitative synthesis approaches. This systematic review was registered on PROSPERO under registration no. CRD420251164201. The study evaluated the incidence, clinical presentation, diagnostic features, management strategies, and outcomes of WE occurring in non-alcoholic adult patients after the performance of gastrointestinal procedures, including endoscopic and surgical interventions.

### 2.1 Study design and duration

This extensive systematic review was completed in September 2025.

### 2.2 Selection criteria

The inclusion criteria were determined using the participants, interventions, comparisons, and outcomes (PICO) framework to ensure a structured and systematic approach to selecting relevant studies. Eligible designs included randomized controlled trials (RCTs), cohort studies, and case series (defined as three or more cases with structured descriptive analysis) written in English, providing robust data for analysis.

1. Population Ineligibility
  - Studies involving participants younger than 18 years or explicitly targeting pediatric populations.
  - Research where alcohol use was the primary cause of WE without a gastrointestinal procedural trigger.
  - Studies describing patients who underwent non-gastrointestinal surgeries, as these fall outside the scope of the review.
2. Intervention Irrelevance
  - Studies examining medical or surgical procedures unrelated to the gastrointestinal tract.
  - Research not involving a gastrointestinal procedural trigger (e.g., nutritional deficiency in the absence of a surgical or endoscopic intervention).
3. Outcome Limitation
  - Studies that did not report clinical presentation, diagnosis, management, or patient outcomes of WE.
  - Articles lacking extractable data relevant to the systematic review objectives.
4. Study Design and Reporting
  - Non-empirical publications, including systematic reviews, meta-analyses, narrative reviews, literature reviews, case reports (defined as publications describing one or two patients without systematic data collection), conference abstracts, expert opinions, or commentaries.
  - Research published in languages other than English, as translation resources will not be utilized.
5. Temporal and Accessibility Constraints
  - Studies published prior to January 2000, as the review aims to capture contemporary evidence.
  - Articles without accessible full-text versions, including abstract-only publications.

By applying these exclusion criteria, the review exclusively focused on high-quality, relevant studies that directly addressed the research objectives and provided robust, actionable insights into the incidence, clinical presentation, diagnosis, management, and outcomes of WE in non-alcoholic adults following gastrointestinal procedures.

### 2.3 Information sources

A systematic search on PubMed (MEDLINE), Scopus, Web of Science, and Embase was conducted, covering 25 years of literature.

### 2.4 Search strategy

A systematic search was conducted across the four databases using keywords and Medical Subject Headings (MeSH) terms. The search included studies published between 1 January 2000 and 1 July 2025, focusing on WE in non-alcoholic adults following gastrointestinal procedures, including both surgical and endoscopic interventions.

The search strategy was as follows: (“Wernicke encephalopathy” OR “Wernicke’s encephalopathy” OR “thiamine deficiency”) AND (“gastrointestinal” OR “bariatric” OR “gastrectomy” OR “bowel resection” OR “intestinal bypass” OR “cystogastrostomy”) AND (“case report” OR “case series” OR “observational” OR “cohort” OR “retrospective” OR “prospective”).

### 2.5 Study selection

Rayyan (QCRI) was used to identify duplicates in the search output. Two independent researchers then applied a set of inclusion/exclusion criteria to screen the combined search results, first by title and abstract, then by full text, to assess relevance. Each manuscript meeting the inclusion criteria underwent thorough evaluation by the two independent reviewers. Discrepancies between reviewers were resolved through discussion, and if necessary, a third reviewer was consulted to reach a final decision.

### 2.6 Strategy for data synthesis

Summary tables were created to provide a qualitative overview of the findings and study components.

### 2.7 Risk of bias assessment

Two researchers independently evaluated the quality of the included studies. Assessment tools were selected based on study design to minimize bias. The Newcastle–Ottawa Scale (NOS) was used for cohort studies to assess methodological quality across three main areas: selection of study groups, comparability between groups, and outcome assessment. For case series, the Joanna Briggs Institute (JBI) Critical Appraisal Checklist was used to evaluate potential biases across 10 domains, including clarity of inclusion criteria, validity of diagnostic methods, consecutive patient inclusion, completeness of demographic and clinical reporting, appropriateness of statistical analysis, and adequacy of follow-up.

## 3 Results

**Figure 1:**
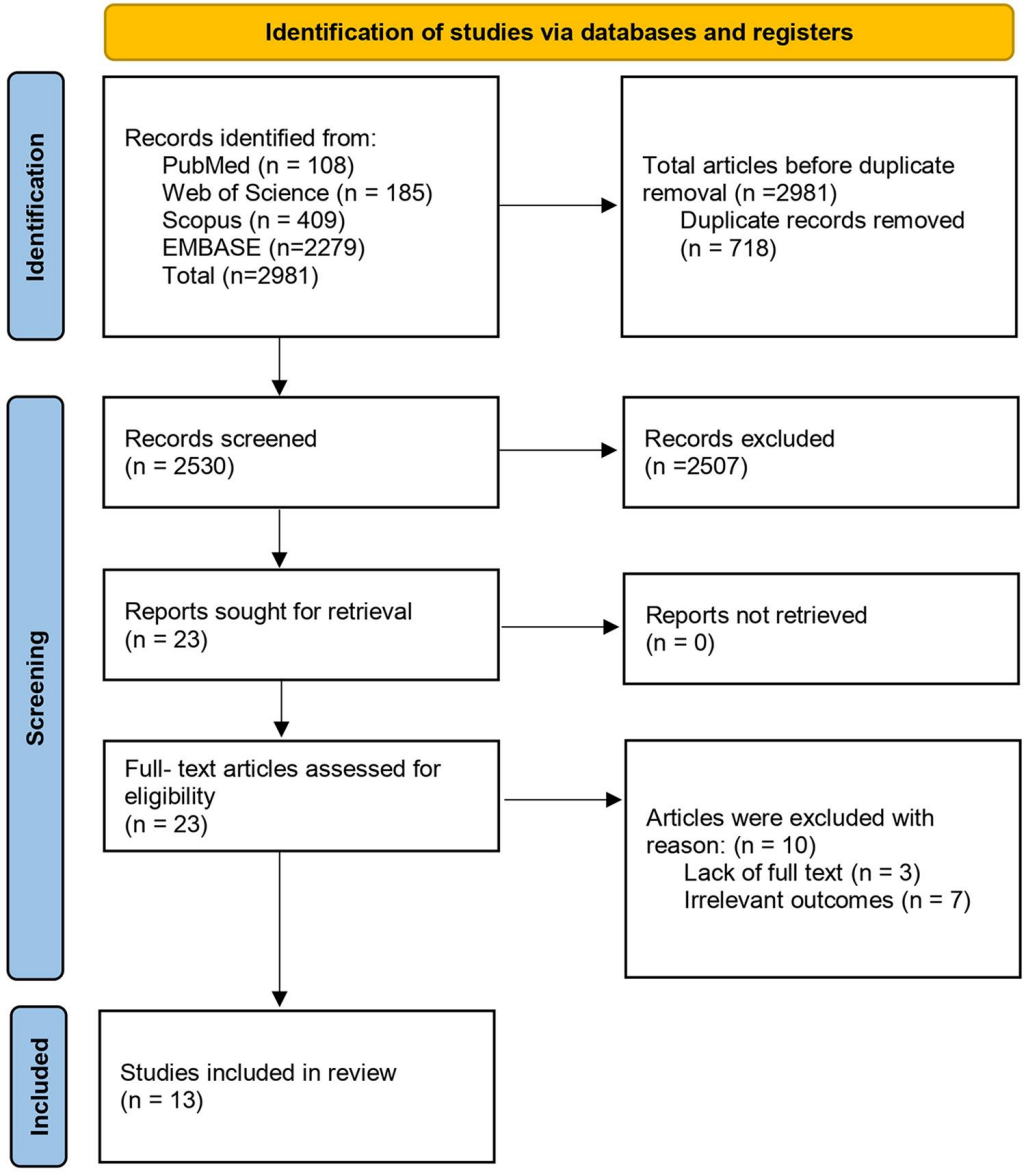
PRISMA flow diagram.

As shown in Figure 1, a total of 2,981 records were identified through database searches, including PubMed, Scopus, and EMBASE. After removing 718 duplicates, 2,530 records were screened by title and abstract. Of these, 2,507 records were excluded because of irrelevance to the research question. The remaining 23 full-text articles were assessed for eligibility. Following full-text review, 10 studies were excluded for the following reasons: conference abstracts missing full text and irrelevant outcomes. Finally, 13 studies met the inclusion criteria and were included in the systematic review. The reported number of participants reflects the total study population; however, not all participants developed Wernicke encephalopathy, as many studies included broader surgical cohorts.

### 3.1 General characteristics of included studies

Across the 13 included studies, patient characteristics varied widely depending on the type of gastrointestinal surgery and the clinical setting (Table 1).

**Table 1:** Study and patient characteristics of postoperative Wernicke encephalopathy.

Author	Year	Country	Journal	Study design	Total participants	Mean age (years)	GI procedure(s)	Preop nutrition	Preop thiamine	Postop fasting	Time to WE onset
Ogershok et al.	2002	USA	*Am J Med Sci*	Retrospective cohort	12	50 ± 17	Bariatric procedures	Poor nutrition; hypoalbuminemia	NR	NR	Variable
Sullivan et al.	2006	USA	*Neuro-Ophthalmology*	Case series	3	36.7	Roux-en-Y; gastroplasty	NR	NR	NR	6 wk–18 mo
Ba et al.	2010	Canada	*Rev Neurol Dis*	Case series	3	41.7	Roux-en-Y; fundoplication	NA	NA	2 d fasting (cases 1,3)	2–3 mo
Jung et al.	2010	Korea	*Cancer Res Treat*	Case series	9	57.4	Sub/total gastrectomy; stent	Malnutrition, wt loss 12–20 kg	7–40 ng/mL (some)	NR	45 d–6 mo
Rufa et al.	2011	Italy	*Int J Neurosci*	Retrospective cohort	10	65.2	Major oncologic resections (GEA, PD, APR, etc.)	Wt loss 5–20 kg; anemia	NR	≥2 wk TPN in 8/10	1 wk–12 mo
Skogar et al.	2015	Sweden	*Elsevier*	Retrospective cohort	359	43 ± 10	RYGB	NR	NR	Prolonged vomiting common	Mean 82 d
Tabbara et al.	2016	France	*Springer*	Retrospective cohort	592	38.1 ± 11.9	Sleeve gastrectomy	Deficiencies in vitamins B1, B6, B9, B12, D	Normal	Severe dysphagia; wt loss	4.6 ± 3.4 mo
Gungor Dogan et al.	2018	Turkey	*Noropsikiyatri Arsivi*	Retrospective cohort	6	39	Carcinoma, pyloric stenosis, colectomy, bypass, and sleeve	All malnourished, wt loss 5–35 kg	NR	NR	1 d–2 mo
Tuncalı et al.	2018	Turkey	*Turk J Anaesthesiol Reanim*	Retrospective cohort	3	26 (21–34)	Sleeve gastrectomy	NR	12–19 µg/L (low)	Vomiting	14–30 d
Yin et al.	2019	China	*SAGE Journals*	Retrospective cohort	17	52.2	GI surgery (varied)	All dietary deficient	Deficiency assumed	NR	NR
Alligier et al.	2020	France	*Elsevier*	Retrospective cohort	12	39 (26–51)	Sleeve gastrectomy; RYGB; OAGB	Only 58% supplemented	NA	NR	Median 6 mo
Gutiérrez-Rey et al.	2024	Colombia	*J Pers Med*	Case series	3	27 (22–30)	Sleeve gastrectomy	All malnourished; micronutrients low	NR	NR	17 d–5 wk
Procaci et al.	2025	Brazil	*Arq Neuro-Psiquiatr*	Retrospective cohort	7	37.7 (27–54)	Bariatric, colectomy, and trauma surgery	All received TPN	NR	TPN 7 d–3 mo	1–12 wk

WE, Wernicke encephalopathy; GI, gastrointestinal; RYGB, Roux-en-Y gastric bypass; OAGB, one-anastomosis gastric bypass; GEA, gastroesophageal anastomosis; PD, pancreaticoduodenectomy; APR, abdominoperineal resection; TPN, total parenteral nutrition; Preop, preoperative; Postop, postoperative; wt, weight; NR, not reported; NA, not available.

Bariatric cohorts included younger patients (mean ages 26–50 years) with vomiting and dysphagia as the major precipitating factors (22, 23, 25, 26, 28, 31). In contrast, oncologic and mixed surgical cohorts featured older patients with a greater malnutrition burden, who were often on total parenteral nutrition (6, 16, 27, 32).

In one of the earliest reports (22), 12 patients in the United States who had undergone bariatric procedures were described. Most of these patients were poorly nourished and exhibited hypoalbuminemia before surgery. The timing of WE was inconsistent, occurring at variable intervals after surgery. Similarly (23), another US study presented three younger patients (average age: 36.7 years) who developed WE between 6 weeks and 18 months postsurgery following Roux-en-Y and gastroplasty.

A Canadian study (24) reported three cases of WE following Roux-en-Y or fundoplication. Two of these patients, who had fasted for 2 days postoperatively, developed WE, highlighting the rapid onset of thiamine depletion. Another study (16) described nine gastric cancer patients undergoing gastrectomy or stent placement. These patients experienced significant weight loss (12–20 kg), documented thiamine deficiency (7–40 ng/mL), and had an onset of WE ranging from 45 days to 6 months.

An Italian (6) study reported 10 older patients (mean age 65 years) who developed WE after major oncologic surgeries. Most of these patients suffered from severe malnutrition and anemia. Eight required prolonged TPN, and the onset of WE ranged from 1 week to 12 months. In contrast, a larger cohort study (25) of 359 Roux-en-Y patients (mean age 43 years) identified vomiting as the main trigger, with a mean onset of 82 days.

Subsequent studies focused on sleeve gastrectomy. A French cohort study (26) (*n* = 592, mean age 38 years) reported frequent preoperative vitamin deficiencies and identified dysphagia and weight loss as key triggers, with onset at approximately 4.6 months. Another study (27) described six malnourished patients after varied procedures, with a weight loss of 5–35 kg and onset between 1 day and 2 months. A Turkish study (28) reported three young patients with low serum thiamine (12–19 µg/L), where vomiting led to onset within 2–4 weeks.

Further reports showed similar trends across diverse procedures, with nutritional deficiency and TPN dependence as commonly noted features across different surgical contexts (29–32).

Collectively, these findings indicate that the risk window for developing WE is much broader than the often cited 4–12-week range, with onset ranging from as early as 14 days postoperatively (28) to as late as 18 months (23).

### 3.2 Clinical presentation and diagnostic features of postoperative Wernicke encephalopathy

The clinical presentation of postoperative WE was variable across all included studies (Table 2). While most studies reported some combination of the classic triad of confusion, ataxia, and oculomotor abnormalities, the complete triad was the exception rather than the rule. Atypical and incomplete presentations were more commonly observed across the variety of procedures.

**Table 2:** Clinical presentation and diagnostic features of postoperative Wernicke encephalopathy.

Study	Thiamine level measured/deficiency	Caine criteria used	MRI findings reported	% MRI positive/characteristic findings	Diagnostic method(s)	Time to onset	Key clinical features
Ogershok (22)	Not routinely measured	No	MRI in 3, CT in 7, autopsy in some	MRI often normal/variable	Clinical triad + imaging + response to thiamine	Variable	Confusion (100%), ataxia (50%), nystagmus (83%)
Sullivan (23)	1 case: 28 nmol/L (low)	No	MRI in Case 1	Positive in symptomatic case	MRI + clinical + EEG	6 wks–18 mo	Vomiting, diplopia, nystagmus, and gait ataxia
Ba (24)	Not available	No	Not reported	NR	NCS + clinical	2–3 months	Diplopia, vertigo, neuropathy, and confusion
Jung (16)	7–40 ng/mL in some cases	No	Positive in all imaged cases	Suggestive MRI changes	MRI + clinical examination	45 d–6 mo	Ophthalmoplegia, ataxia, confusion, and seizures
Rufa (6)	Not reported	Yes (Caine applied)	Thalamus, mammillary bodies, and periaqueductal changes	MRI supportive in most	Caine criteria + MRI	1–12 months	Mental status change (100%), oculomotor signs (100%)
Skogar (25)	Not reported	Not specified	Not detailed	Clinical diagnosis predominant	Clinical + response to thiamine	∼82 days	Triad + vomiting
Tabbara (26)	Normal preop thiamine	No	MRI + EMG used	Not quantified	MRI + neuro exam	4.6 ± 3.4 months	Fatigue, ataxia, dysphagia, and neuropathy
Gungor Dogan (27)	Presumed deficient	Yes (clinical criteria used)	MRI in all cases	100% MRI supportive	MRI + EEG + cognitive testing	1 day–2 months	4/6 full triad; vomiting common
Tuncali (28)	12–19 µg/L (low in all)	Not reported	Classic MRI lesions in all	100%	MRI + clinical	14–30 days	Confusion, nystagmus, and ataxia
Yin (29)	Not quantitatively measured	Yes	MRI used in all	Not specified	Caine criteria + MRI	Not specified	AMS 100%, eye signs 29%, and ataxia 6%
Alligier (30)	Not reported	Not reported	MRI/CT in 18/22	Majority positive	Clinical + imaging	Median 6 months	Triad present in all
Gutiérrez-Rey (31)	Malnutrition/micronutrient deficiency present	Not reported	MRI + NCS	3/3 cases supportive	MRI + clinical + NCS	17 d–5 wks	Nystagmus, amnesia, and ophthalmoplegia
Procaci (32)	Not reported	Not reported	MRI supportive in most	Not quantified	Clinical + MRI	1–7 weeks	Ataxia (100%), nystagmus (6/7)

WE, Wernicke encephalopathy; MRI, magnetic resonance imaging; CT, computed tomography; EEG, electroencephalography; EMG, electromyography; NCS, nerve conduction studies; MB, mammillary bodies; PAG, periaqueductal gray; NR, not reported.

In 2002, a cohort from the United States described 12 patients, all of whom developed confusion. Nystagmus occurred in 83% of patients and ataxia in 50%, with several progressing to coma. Diagnosis was based on clinical examination and, in some cases, MRI, CT, or autopsy. Long-term follow-up showed frequent residual neurological deficits (22).

In 2006, a case series from the United States (23) reported three patients with distinct symptom patterns. The first presented with vomiting, gait ataxia, and nystagmus; the second with diplopia, ptosis, and gaze palsy; and the third with persistent emesis. Diagnosis was confirmed using MRI, thiamine levels, electroencephalography (EEG), and clinical findings. Only one patient had a long-term follow-up of 2.5 years, revealing mild residual symptoms. In 2010, a Canadian study (24) described three patients presenting with diplopia, vertigo, confusion, and neuropathic symptoms including pain and weakness. One patient also developed bradykinesia. Nerve conduction studies were essential for diagnosis, and follow-up, ranging from 6 months to 6 years, highlighted chronic sequelae.

A Korean case series (16) studied nine patients, five of whom displayed the full triad. Other patients developed seizures, dizziness, or gaze palsy. Diagnosis was made using MRI and neurological examination, although follow-up details were not provided. In 2011, an Italian cohort (6) described 10 patients: All developed mental status changes and oculomotor signs, half experienced ataxia, and 60% showed polyneuropathy. The Caine criteria were used in combination with MRI. Outcomes were variable, with both full and partial recoveries.

Beyond the classical triad, atypical features were prominently noted across multiple cohorts, with larger cohorts offering broader clinical pictures. In 2015, a large Swedish cohort study (25) described patients presenting with confusion, ophthalmoplegia, and ataxia, as well as fatigue and diplopia. Diagnosis relied on clinical assessment, imaging, and response to thiamine. A French cohort (26) reported additional symptoms such as memory loss, paresthesia, vomiting, dysphagia, and motor deficits. Diagnosis was confirmed by neurology consultations, MRI, and electromyography (EMG), and symptoms generally resolved within months.

A Turkish cohort (27) reported six cases, four of which exhibited the full triad. Vomiting was a frequent trigger, and cognitive deficits were noted. Diagnosis involved MRI, EEG, and cognitive testing; one patient died during follow-up. In the same year, another Turkish study (28) described three patients with confusion, nystagmus, ophthalmoplegia, and altered consciousness. MRI changes in the thalamus, mammillary bodies, and periaqueductal gray supported the diagnosis, and all recovered within weeks.

A cohort from China (29) reported altered mental status in every patient, oculomotor signs in 29%, and gait ataxia in only 6%, demonstrating the common occurrence of incomplete presentations. Meanwhile, a French cohort study (30) found at least one feature of the triad in every patient, with frequent reports of vomiting; imaging confirmed the majority of diagnoses. A Colombian case series (31) described three patients, all with nystagmus, while additional findings included ophthalmoplegia, amnesia, and neuropathic pain. MRI and nerve conduction studies confirmed diagnoses, with one case followed up for a year. Most recently, Procaci et al. (32) reported seven patients, all of whom experienced ataxia, six developed nystagmus, and several developed neuropathy, tremor, and dysarthria. MRI confirmed their diagnoses, and follow-up extending up to 17 years revealed persistent neurological deficits in some.

In summary, confusion and ocular abnormalities were the most consistent features, although complete triad presentations were less common. This was illustrated by Yin et al. (29), who reported that altered mental status occurred in 100% of patients but oculomotor signs were present in only 29% and ataxia in 6%.

WE remains, for the most part, a clinical diagnosis. MRI, when positive, particularly with FLAIR sequences, provides strong corroborating evidence. The characteristic changes are most commonly noted in the thalamus, mammillary bodies, and periaqueductal gray (16, 27, 28). However, given its sensitivity of approximately 50%–60%, a normal MRI does not exclude WE, and treatment should never be delayed pending imaging results. Whole blood thiamine levels may support diagnosis, whereas serum thiamine levels are not reliable due to variability in tissue distribution. Long-term follow-up further confirmed that residual deficits remained common even after treatment.

### 3.3 Management and outcomes of postoperative Wernicke encephalopathy

Treatment strategies were based on parenteral thiamine replacement, although considerable variations in exact dosing and duration existed among the studies (Table 3). Dosing ranged from 100 mg/day (16) to 300–500 mg/day (6), 1 g/day (26), and 500 mg three times daily (31), demonstrating a lack of a standard protocol.

**Table 3:** Management and outcomes of postoperative Wernicke encephalopathy.

Study	IV Thiamine dose	Duration of IV therapy	Taper/oral transition	Additional vitamins	Treatment strategy	Recovery	Mortality
Ogershok (22)	IV thiamine (dose NR)	NR	Oral maintenance	Multivitamins	Empiric replacement	Partial recovery in most	4/12
Sullivan (23)	100 mg IV	Acute + prolonged (3 months in some)	Oral thiamine 50 mg/day	Multivitamins, B12	Long-term supplementation	Mostly full recovery	0
Ba (24)	IV thiamine (NR)	NR	Oral B12/gabapentin in some	B12 and multivitamins	Supportive + supplementation	Partial/variable	0
Jung (16)	100 mg/day IV	4–17 days	Oral continuation	NR	High-dose early therapy	7 improved	2
Rufa (6)	300–500 mg/day IV/IM	Variable	Oral in survivors	NR	High-dose parenteral therapy	3 full and 3 partial	4
Skogar (25)	IV thiamine (dose NR)	NR	Oral support	Electrolytes	Nutritional + vitamin therapy	Mixed outcomes	1
Tabbara (26)	1 g IV	Days–weeks	Oral maintenance	B6, B12, and multivitamins	Aggressive supplementation	Mostly improved	0
Gungor Dogan (27)	≥500 mg/day IV	NR	Oral maintenance	Multivitamins	High-dose replacement	2 recovered	1
Tuncali (28)	500 mg/day IV	Days until recovery	Switched to oral	Supportive care	Early aggressive therapy	Full recovery (all)	0
Yin (29)	200–500 mg IV TID	Until response	Oral continuation	NR	Early vs. delayed therapy comparison	14 recovered	0
Alligier (30)	Vitamin B1 (76% received)	NR	Not specified	B6, B12, and B3	Variable supplementation	9 recovered	2
Gutiérrez-Rey (31)	500 mg IV q8h → taper	3–14 days IV + prolonged oral	Long-term oral (weeks–1 year)	B12 and folate	High-dose structured regimen	3/3 recovery	0
Procaci (32)	300 mg/day IV	7 days–3 months (TPN dependent)	Oral/maintenance in some	B12, folate	Nutritional + vitamin replacement	2 improved and 5 poor	0

WE, Wernicke encephalopathy; IV, intravenous; IM, intramuscular; PN, parenteral nutrition; B1, vitamin B1 (thiamine); B6, vitamin B6 (pyridoxine)l B12, vitamin B12 (cobalamin); B3, vitamin B3 (niacin); TID, three times daily; NR, not reported; d, days; y, years; mo, months.

In 2002, a cohort from the United States (22) treated 12 patients with IV thiamine followed by oral maintenance. Only two patients fully recovered, while several developed lasting deficits, and four died. In a 2006 case series (23), patients received IV thiamine, 3 months of parenteral supplementation, and oral continuation. All three patients recovered, although some experienced mild residual symptoms.

Some cohorts recorded variable recovery, wherein some patients had persistent neurological symptoms, while others died (16, 24). An Italian cohort conducted in 2011 (6) administered 300–500 mg/day of thiamine via the IV or IM route. However, three patients recovered completely, three partially, and four died, reflecting the poor prognosis of older, malnourished cancer patients.

Outcomes were generally better in bariatric cohorts. Skogar et al. (25) administered IV thiamine, electrolytes, and nutritional support. Three patients achieved complete recovery, six had residual deficits, and two were left disabled. However, only one death occurred among 359 patients. Tabbara et al. (26) treated patients with 1 g IV thiamine along with B6, B12, and parenteral nutrition. Most patients improved, some with residual deficits, and no deaths were reported among 592 cases.

Gungor Dogan et al. (27) prescribed ≥500 mg/day of IV thiamine followed by oral dosing. Two patients recovered, three had residual symptoms, and one died. Tuncalı et al. (28) administered 500 mg/day of IV thiamine with supportive care and oral continuation in three young patients, all of whom recovered completely. A Chinese cohort from 2019 (29) administered 200–500 mg of IV thiamine three times daily; 14 of 17 patients recovered, but three were left with deficits, and two developed Korsakoff syndrome. Notably, they also reported shorter hospital stays for patients receiving thiamine (12.2 vs. 36.8 days) (29).

More recent studies similarly reported mixed outcomes, with patient recovery varying from complete to poor, and persistent deficits documented even in patients who survived (30–32).

Overall, early initiation of high-dose IV thiamine was the key determinant of outcome. Rufa et al. (6) prominently illustrated this by documenting a 40% mortality rate in older oncologic patients despite daily doses of 300–500 mg. In contrast, Skogar et al. (25) demonstrated only one death among 359 bariatric patients (0.3%). This difference reflects not the dosing itself, but rather patient age, malnutrition burden, and likely delayed recognition in oncologic settings. Mortality was highest in older, nutritionally depleted oncologic patients, while promptly treated bariatric cohorts had far lower mortality but still experienced lasting neurological impairment in a proportion of cases.

### 3.4 Mortality in patients with Wernicke encephalopathy following gastrointestinal procedures

Mortality among patients with Wernicke encephalopathy following gastrointestinal procedures varied widely across studies, ranging from 0.3% to 40% (6, 25). This variability likely reflects differences in study populations, surgical indications, baseline nutritional status, and timing of intervention.

### 3.5 Risk of bias assessment

**Figure 2:**
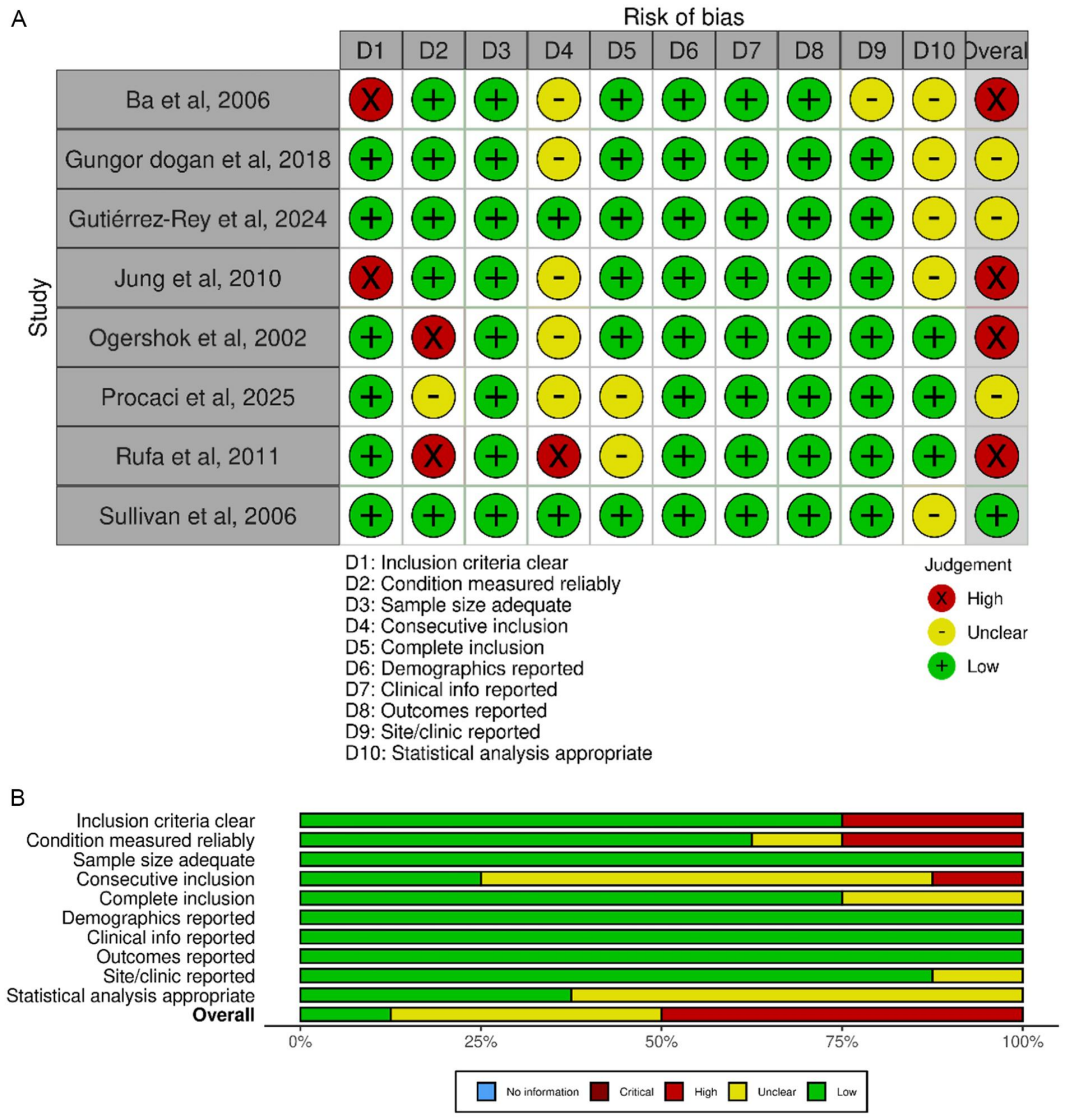
**(A)** Risk of bias traffic plot for case series, assessed across 10 domains using the JBI tool. **(B)** Summary plot showing the distribution of judgments (high, low, unclear) across all included case series.

The Joanna Briggs Institute (JBI) checklist was used for the appraisal of case series, and the Newcastle–Ottawa Scale (NOS) was applied to cohort studies. Most case series demonstrated a low risk of bias in domains related to case definition and the validity of diagnostic methods, indicating that WE was generally identified with reasonable accuracy (Figure 2, Supplementary Table 1A). However, several limitations were noted, particularly incomplete follow-up data and the absence of information on whether patients were recruited consecutively. These methodological gaps are characteristic of case series, which tend to emphasize descriptive reporting over systematic rigor.

**Figure 3:**
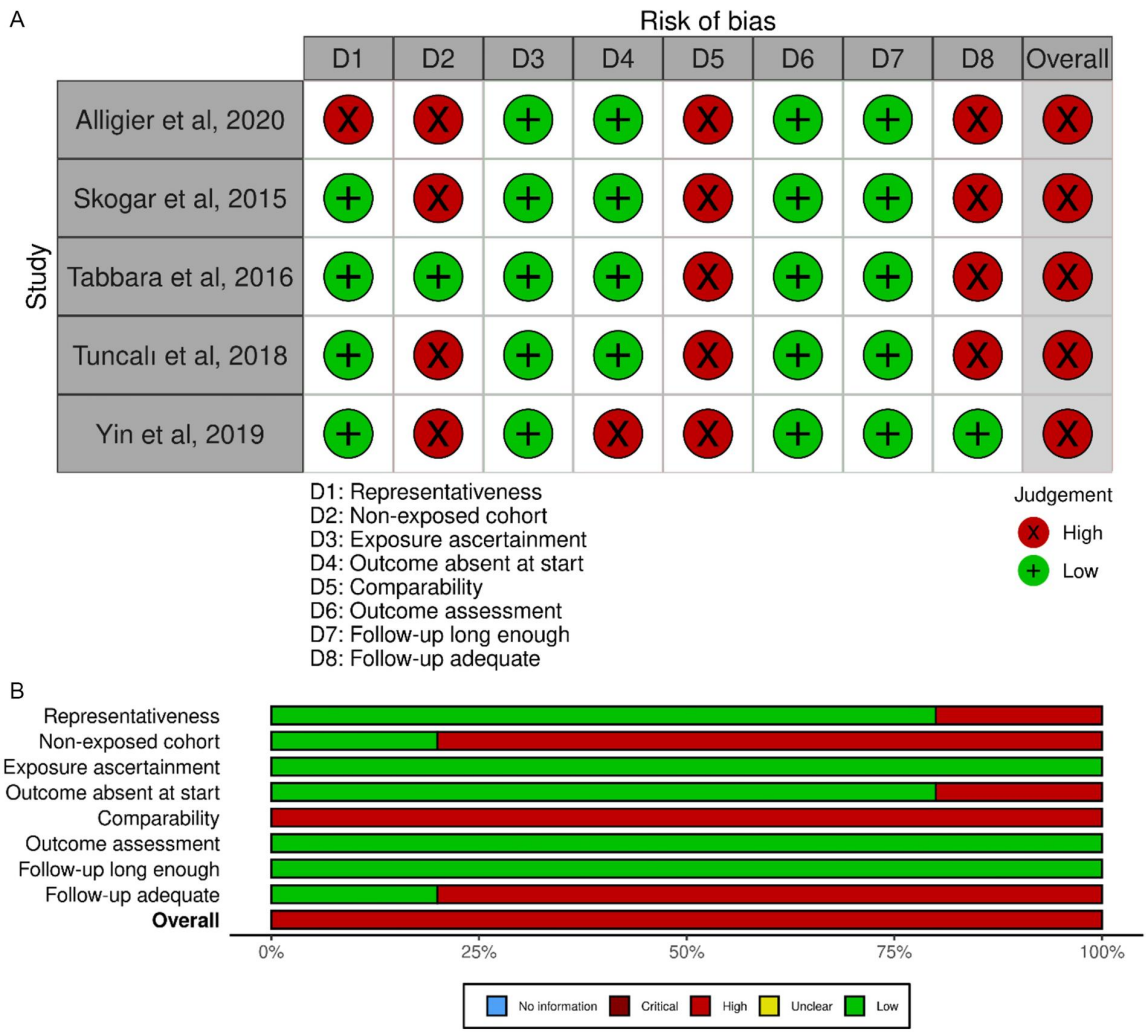
**(A)** Risk of bias traffic plot for cohort studies, assessed across eight domains using the NOS tool. **(B)** Summary plot showing the distribution of judgments (high or low) across all included cohort studies.

The cohort studies were assessed as being of moderate to low quality (Figure 3, Supplementary Table 1B). Strengths included adequate representativeness of the exposed cohort, reliable ascertainment of surgical exposure, and objective outcome assessment. The main weaknesses were in the comparability of cohorts and adequacy of follow-up. Several studies failed to adjust for baseline nutritional deficiencies or comorbid conditions that could act as confounders. Despite these limitations, the cohort data overall provided sufficiently robust evidence to identify consistent postoperative risk factors and temporal patterns for WE.

## 4 Discussion

This systematic review included 13 studies that highlighted the clinical significance of WE following gastrointestinal surgeries, particularly bariatric and oncologic procedures. WE was consistently associated with nutritional deficiencies, especially thiamine depletion, which may be exacerbated by vomiting (25, 28), prolonged fasting, and inadequate supplementation. Symptoms appeared anywhere from 1 day to more than a year after surgeries, suggesting that WE can present both acutely and gradually.

Of the classical triad of confusion, ataxia, and oculomotor dysfunction, confusion and ocular signs were the most frequently observed features. However, many patients presented with incomplete or unusual symptoms, which challenged prompt diagnosis. MRI was the most common diagnostic tool (16, 26), revealing characteristic changes in the thalamus, mammillary bodies, and periaqueductal gray, underscoring its significance in confirming WE. However, MRI sensitivity is limited (approximately 50%–60%), and a normal MRI does not exclude the diagnosis. CT imaging, in contrast, is often normal and has limited sensitivity for detecting WE. Many patients experienced long-term neurological consequences even with treatment, underlining the need for early detection. Treatment approaches focused on intravenous thiamine, with considerable variability in dosing regimens. Standard dosing (e.g., 100 mg/day) was commonly reported, whereas high-dose therapy typically exceeded 500 mg/day to 1 g/day intravenously frequently combined with additional B vitamins (24, 26, 30) and nutritional support (25). Detailed case-level treatment regimens are provided in Supplementary Table 2.

Outcomes differed significantly: Bariatric cohorts generally had better recovery rates and lower mortality, likely due to their younger age and earlier intervention. In contrast, oncology patients, especially older individuals with significant malnutrition, experienced worse outcomes and higher mortality rates. The review found that early initiation of thiamine therapy was the most critical determinant of recovery. Delayed treatment was linked to persistent deficits, Korsakoff syndrome (29), and death in several cases (6, 22). Mortality rates ranged from 0.3% in a large cohort (25) to 40% in a smaller series (Rufa et al.) (6).

Previous systematic reviews (19, 20) primarily focused on bariatric surgery, especially Roux-en-Y gastric bypass, identifying common triggers such as vomiting, rapid weight loss, and lack of supplements. This systematic review builds on existing literature by examining WE following a broader range of gastrointestinal procedures in non-alcoholic patients. Unlike prior reviews that primarily focused on bariatric surgery, our analysis includes diverse surgical contexts such as oncologic and emergency procedures. The findings reinforce previously identified triggers, including vomiting and malnutrition, while demonstrating that WE is not limited to bariatric populations but can occur across a wide spectrum of gastrointestinal surgeries.

Mortality varied considerably across the included studies, underscoring the seriousness of WE despite advances in diagnosis and management. Outcomes appeared more favorable in bariatric populations, in whom structured supplementation and earlier recognition are more routinely implemented. In contrast, higher mortality was observed among older, malnourished oncologic patients, likely reflecting delayed diagnosis and inconsistent nutritional support (6).

Compared with previous systematic reviews, this analysis highlights a broader deficiency in perioperative thiamine assessment. Concerns have been raised regarding the reliability of plasma thiamine as a diagnostic indicator, particularly as only a limited number of included studies assessed serum or erythrocyte thiamine levels. Moreover, serum thiamine levels may not accurately reflect true deficiency and can remain within normal ranges even in symptomatic patients. WE remains primarily a clinical diagnosis, and treatment should not be delayed for confirmatory testing. Furthermore, the heterogeneity in dosing regimens, ranging from 100 mg/day to >1 g/day intravenously, shows the absence of standardized treatment protocols across surgical specialties. Our findings confirm that the risk of WE extends beyond bariatric surgery and that present preventive strategies are insufficiently generalized across GI surgical practice.

This systematic review has several strengths that contribute to its value. First is the overarching and contemporary search strategy. The review systematically searched four major databases: PubMed (MEDLINE), Scopus, Web of Science, and Embase, covering a 25-year period (2000–2025). This extensive temporal and database range improved the chances of capturing all studies related to WE in non-alcoholic adults after gastrointestinal surgeries. The inclusion of both surgical and endoscopic procedures guaranteed that the review encompassed the entire clinical range of gastrointestinal procedure-related WE. The use of standardized critical appraisal tools represents another key advantage. Cohort studies were assessed with the Newcastle–Ottawa Scale (NOS), while case series were evaluated using the Joanna Briggs Institute (JBI) Critical Appraisal Checklist. This dual-instrument approach enabled a thorough and design-focused quality assessment, increasing the reliability of the conclusions. Excluding non-empirical studies and publications before the year 2000 improved the review by ensuring that the evidence reflected current diagnostic standards, surgical techniques, and nutritional management strategies, making the findings more applicable to today's practice.

Several limitations should be acknowledged. First, despite an extensive search strategy, limiting the review to English-language studies may have led to language bias, possibly omitting important research published in other languages. The predominance of retrospective cohorts and case series introduces a significant risk of bias, limiting the strength and generalizability of the findings. Furthermore, focusing only on freely accessible full-text studies poses a potential limitation, as it may have excluded pertinent evidence found in subscription-based journals, thus making the literature synthesis incomplete. Another limitation arises from the inherent methodological weaknesses of the included study designs. The majority of available evidence in this field typically comes from retrospective cohort studies or case series, which are susceptible to selection bias, incomplete data reporting, and a lack of standardized follow-up. These factors may affect the accuracy and generalizability of the synthesized findings.

Clinicians need to understand that WE can occur following any major gastrointestinal treatment, especially in patients who are dependent on total parenteral nutrition, experience postoperative vomiting, or have been fasting for an extended period. All high-risk patients should begin empirical thiamine supplementation immediately rather than waiting for laboratory confirmation, as the effectiveness of intervention heavily relies on its promptness, as evidenced by Patterson et al. (33). This approach should be supported by integrating standardized thiamine supplementation regimens and routine perioperative micronutrient assessments, which are well established in bariatric surgery, into the treatment protocols of patients undergoing all major GI operations. To guarantee early detection, timely treatment, and avoidance of irreparable neurological consequences, it is also crucial to raise awareness among surgeons, anesthesiologists, and nutritionists regarding the frequently unusual, non-alcoholic manifestations of WE.

To better assess the actual incidence of WE after various GI surgeries, future research should focus on prospective data. Existing research is mainly retrospective and case series, hence limiting both its relevance and ability to identify causal factors. To address these gaps, future studies should use standardized diagnostic criteria that include radiological and clinical data, thereby enabling more consistent reporting and reliable systematic reviews. Patients undergoing enteral or parenteral feeding are at a high risk of thiamine deficiency. For those patients specifically, future research should determine the optimal timing, dose, and duration of thiamine supplementation.

## 5 Conclusion

WE following gastrointestinal procedures presents significant diagnostic challenges because of its variable and often atypical presentation. MRI can support the diagnosis when positive, but a normal result does not rule out WE and should never delay the initiation of intravenous thiamine. Given these challenges, early recognition and prompt thiamine administration are essential. Prophylactic thiamine supplementation should be strongly considered in high-risk patients, particularly those with malnutrition, prolonged fasting, or persistent vomiting.

## Data Availability

The original contributions presented in the study are included in the article/Supplementary Material, and further inquiries can be directed to the corresponding author.
