# Supplementary material for: Wernicke encephalopathy in non-alcoholic patients following gastrointestinal procedures: a systematic review
Source: Front Surg. 2026 Jun 30;13:1802386. doi: 10.3389/fsurg.2026.1802386 (PMC13365056; doi:10.3389/fsurg.2026.1802386)
Supplement: Supplementary file 1 [file Table1.docx]

**Supplementary Material**

**Supplementary Table 1A. Risk of bias assessment of case series using Joanna Briggs Institute (JBI) checklist**

| **Study** | **Clear inclusion criteria** | **Consecutive inclusion** | **Valid diagnosis** | **Demographics reported** | **Clinical details complete** | **Follow-up adequate** | **Outcome reporting clear** | **Overall risk** |
| --- | --- | --- | --- | --- | --- | --- | --- | --- |
| Sullivan et al., 2006 | Yes | Unclear | Yes | Yes | Yes | No | Yes | Moderate |
| Ba et al., 2010 | Yes | No | Yes | Yes | Yes | Unclear | Yes | Moderate |
| Jung et al., 2010 | Yes | Unclear | Yes | Yes | Partial | No | Yes | Moderate |
| Gutiérrez-Rey et al., 2024 | Yes | Yes | Yes | Yes | Yes | Partial | Yes | Low–Moderate |

**Supplementary Table 1B. Methodological quality assessment of cohort studies using the Newcastle–Ottawa Scale (NOS)**

*(Maximum score = 9 stars)*

| **Study** | **Selection (4★)** | **Comparability (2★)** | **Outcome (3★)** | **Total NOS Score** | **Overall Quality** |
| --- | --- | --- | --- | --- | --- |
| Ogershok et al., 2002 | ★★★★ | ★★ | ★★ | 7/9 | High–Moderate |
| Rufa et al., 2011 | ★★★ | ★ | ★★ | 6/9 | Moderate |
| Skogar et al., 2015 | ★★★★ | ★ | ★★ | 6/9 | Moderate |
| Tabbara et al., 2016 | ★★★★ | ★★ | ★★★ | 9/9 | High |
| Güngör Doğan et al., 2018 | ★★★ | ★ | ★★ | 5/9 | Moderate |
| Tuncalı et al., 2018 | ★★★ | ★ | ★★ | 5/9 | Moderate |
| Yin et al., 2019 | ★★★ | ★ | ★★ | 5/9 | Moderate |
| Alligier et al., 2020 | ★★★★ | ★★ | ★★ | 8/9 | High |
| Procaci et al., 2025 | ★★★ | ★ | ★★ | 5/9 | Moderate |

**NOS interpretation:**

- 7–9 = High quality
- 5–6 = Moderate quality
- ≤4 = Low quality

**Supplementary Table 2**. Detailed thiamine dosing regimens and treatment protocols in post-operative Wernicke encephalopathy (case-level where available)

| **Study** | **Case** | **IV Thiamine Regimen** | **Duration of IV Therapy** | **Transition to Oral Therapy** | **Oral Maintenance Dose** | **Adjunct Therapy** | **Notes** |
| --- | --- | --- | --- | --- | --- | --- | --- |
| Ogershok 2002 | Mixed cohort | IV thiamine (dose not standardized) | Variable (acute hospital phase) | Yes in survivors | Oral thiamine maintenance (NR) | Multivitamins | Some cases diagnosed post-mortem |
| Sullivan 2006 | Case 1 | IV thiamine 100 mg | Acute phase then extended support | Yes | 50–100 mg/day | Multivitamins | 3-month parenteral continuation in some cases |
|  | Case 2 | IV thiamine 100 mg | Acute | Yes | 50 mg/day | Multivitamins | Rapid improvement in ocular symptoms |
|  | Case 3 | IV thiamine 100 mg | Acute | Yes | NR | TPN + thiamine | Persistent nausea pre-treatment |
| Ba 2010 | Case 1 | IV thiamine (dose NR) | Acute | Yes | NR | B12 injections | Residual neuropathy persisted |
|  | Case 2 | Not applicable (no WE) | - | - | - | - | Included as control surgical case |
|  | Case 3 | IV thiamine + B12 | Acute | Yes | B12 maintenance | Vitamin B12 | Long-term neurological deficits |
| Jung 2010 | Mixed cases | IV thiamine 100 mg/day | 4–17 days | Yes | NR | Multivitamins (variable) | Cancer cachexia common |
| Rufa 2011 | Cohort | IV thiamine 300–500 mg/day (often divided doses) | Variable (days–weeks) | Yes | Oral continuation in survivors | Nutritional support | Delayed diagnosis common |
| Skogar 2015 | Cohort | IV thiamine (dose NR) | Acute hospital admission | Yes | Oral supplementation | Electrolytes, nutrition | Vomiting common trigger |
| Tabbara 2016 | Cohort | IV thiamine 1 g/day | Several days to weeks | Yes | Oral maintenance (NR) | B6, B12, trace elements | Aggressive supplementation protocol |
| Gungor Dogan 2018 | Case 1 | IV thiamine ≥500 mg/day | NR | Yes | Oral maintenance | Multivitamins | Malignancy-related malnutrition |
|  | Case 2 | IV thiamine ≥500 mg/day | NR | Yes | Oral maintenance | Multivitamins | Rapid onset (1 day post-op) |
|  | Case 3 | IV thiamine ≥500 mg/day | NR | Yes | Oral maintenance | Multivitamins | Neurological improvement partial |
|  | Case 4 | IV thiamine ≥500 mg/day | NR | Yes | Oral maintenance | Multivitamins | Bariatric surgery case |
|  | Case 5 | IV thiamine ≥500 mg/day | NR | Yes | Oral maintenance | Multivitamins | Sleeve gastrectomy |
|  | Case 6 | IV thiamine ≥500 mg/day | NR | Yes | Oral maintenance | Multivitamins | Poor prognosis case |
| Tuncali 2018 | Case 1 | IV thiamine 500 mg/day | Until clinical recovery | Yes | Oral maintenance | Electrolyte correction | Vomiting-related deficiency |
|  | Case 2 | IV thiamine 500 mg/day | Until recovery | Yes | Oral maintenance | Supportive care | Rapid improvement |
|  | Case 3 | IV thiamine 500 mg/day | Until recovery | Yes | Oral maintenance | Supportive care | Full neurological recovery |
| Yin 2019 | Mixed cohort | IV thiamine 200–500 mg TID | Until response | Yes | Oral continuation | Nutritional correction | Early treatment = better outcome |
| Alligier 2020 | Cohort | Vitamin B1 supplementation (dose variable) | NR | Yes | NR | B6, B12, B3 | Preventive supplementation insufficient |
| Gutiérrez-Rey 2024 | Case 1 | IV thiamine 500 mg q8h × 3 days → 250 mg q8h × 5 days | 8 days IV | Yes | 100 mg/day long-term | B6, B12, folate | Structured tapering protocol |
|  | Case 2 | IV thiamine 500 mg q8h × 14 days | 14 days IV | Yes | 100 mg/day | B12 | Persistent amnesia |
|  | Case 3 | IV thiamine 500 mg q8h × 5 days → taper | 5 days IV | Yes | 100 mg/day | Multivitamins | Full recovery |
| Procaci 2025 | Case 1 | IV thiamine 300 mg/day | 7 days–3 months (TPN dependent) | Yes | Oral maintenance (NR) | B12, folate | Long-term follow-up |
|  | Case 2 | IV thiamine 300 mg/day | Variable | Yes | NR | B12 | Partial recovery |
|  | Case 3 | IV thiamine 300 mg/day | Variable | Yes | NR | B12 | Poor outcome |
|  | Case 4 | IV thiamine 300 mg/day | Variable | Yes | NR | B12 | Trauma-related case |
|  | Case 5 | IV thiamine 300 mg/day | Variable | Yes | NR | B12 | Emergency surgery |
|  | Case 6 | IV thiamine 300 mg/day | Variable | Yes | NR | B12 | Sepsis + colectomy |
|  | Case 7 | Delayed supplementation | Chronic phase | Yes | Long-term oral | B12 | Late initiation (17 years) |
| Skogar 2015 | Cohort | IV thiamine (NR) | Acute phase | Yes | Oral supplementation | Electrolytes | Mortality in 1 case |
